# Biomaterial-based mechanical regulation facilitates scarless wound healing with functional skin appendage regeneration

**DOI:** 10.1186/s40779-024-00519-6

**Published:** 2024-02-18

**Authors:** Ying-Ying Li, Shuai-Fei Ji, Xiao-Bing Fu, Yu-Feng Jiang, Xiao-Yan Sun

**Affiliations:** 1 Research Center for Tissue Repair and Regeneration Affiliated to the Medical Innovation Research Department, Chinese PLA General Hospital and PLA Medical College; PLA Key Laboratory of Tissue Repair and Regenerative Medicine and Beijing Key Research Laboratory of Skin Injury, Repair and Regeneration; Research Unit of Trauma Care, Tissue Repair and Regeneration, Chinese Academy of Medical Sciences, 2019RU051, Beijing, 100048 China; 2 Department of Tissue Regeneration and Wound Repair, Chinese PLA General Hospital, Beijing, 100853 China

**Keywords:** Scarless, Wound healing, Biomaterials, Mechanical cues, Skin appendages

## Abstract

Scar formation resulting from burns or severe trauma can significantly compromise the structural integrity of skin and lead to permanent loss of skin appendages, ultimately impairing its normal physiological function. Accumulating evidence underscores the potential of targeted modulation of mechanical cues to enhance skin regeneration, promoting scarless repair by influencing the extracellular microenvironment and driving the phenotypic transitions. The field of skin repair and skin appendage regeneration has witnessed remarkable advancements in the utilization of biomaterials with distinct physical properties. However, a comprehensive understanding of the underlying mechanisms remains somewhat elusive, limiting the broader application of these innovations. In this review, we present two promising biomaterial-based mechanical approaches aimed at bolstering the regenerative capacity of compromised skin. The first approach involves leveraging biomaterials with specific biophysical properties to create an optimal scarless environment that supports cellular activities essential for regeneration. The second approach centers on harnessing mechanical forces exerted by biomaterials to enhance cellular plasticity, facilitating efficient cellular reprogramming and, consequently, promoting the regeneration of skin appendages. In summary, the manipulation of mechanical cues using biomaterial-based strategies holds significant promise as a supplementary approach for achieving scarless wound healing, coupled with the restoration of multiple skin appendage functions.

## Background

Achieving scarless wound healing with skin appendage regeneration requires the creation of a harmonious scarless environment and the mobilization of resident cells through biochemical or biomechanical cues. Biomechanical cues refer to how cells and tissues respond to both internal and external mechanical forces. One area of research focus has been the utilization of biomaterials to promote cutaneous wound healing, and increasing evidence suggests that biomechanical cues play a significant role in how the skin interacts with these materials. The skin is a highly specialized mechanoresponsive organ that reacts to mechanical forces in various physiological contexts, including growth, obesity, and friction. Skin appendages, such as hair follicles (HFs), sebaceous glands, and sweat glands (SwGs), are essential for skin homeostasis and function [1]. These appendages are constantly exposed to dynamic mechanical cues, from rapid expansion during embryonic and early postnatal development to external forces throughout life. However, significant skin defects caused by burns, severe trauma, or chronic diseases often disrupt the native mechanical homeostasis of skin and lead to the loss of functional cells, including basal keratinocytes, dermal papilla cells, and sweat gland cells (SGCs). The role of mechanical cues in scar formation and wound healing has been gradually revealed [2], which has opened up promising strategies for scarless wound healing [3, 4]. Biomaterials with unique physical properties, such as stiffness, topography, magnetism, and conductivity, have gained attention. To date, biomaterials with specific physical properties can provide a structural framework to facilitate the attachment and migration of host stem and progenitor cells, and drive the differentiation of these cells into tissue-specific cell types by breaking through the epigenetic barrier, improve immune ecology so as to enhance the efficiency of neovascularization, restoration of innervation, and regeneration of skin appendages [5–8]. Exact mechanisms by which biomaterials influence cells and ways to use them to stimulate tissues are interesting. This review outlines recent developments in biomaterial-based mechanical regulation. It begins by discussing the mechanical cue-dependent scarring process, including key factors such as molecules [transforming growth factor-β (TGF-β), vascular endothelial growth factor (VEGF), neuropeptides, etc.] and cells (fibroblasts, keratinocytes, immunocytes, etc.) [9–12]. It then delves into the biophysical characteristics of biomaterials that modulate extracellular signaling, alter cellular epigenetic states, support cell migration, and allow tissue ingrowth. Finally, it explores the potential of biomaterial-based mechanical cues to create scarless environment and to stimulate stem and progenitor cells for regeneration of skin appendages. In particular, by combining the principles of developmental science and biomaterials, we anticipate that biomaterials can facilitate the regeneration of multiple skin appendages through sequential regulation. Overall, this review offers novel strategies based on the physical properties of biomaterials to drive scarless wound healing and skin appendage regeneration.

## Mechanical signal-dependent scarring

Previous study shed light on the effect of mechanical forces on scar formation, and the mechano-transduction signaling pathway can be a potential target to reduce scarring and promote skin regeneration [3]. From clinical observations and laboratory evidences, mechanical forces are involved in the process of wound healing, and regulate scar formation-related factors, including TGF-β, epithelial-mesenchymal transition (EMT), neurovascular abnormalities as well as inflammation [10, 13–16]. A deeper understanding of the mechanical dependence of scarring is conducive to the development of biomaterials for mechanical regulation.

### Clinical observation

Typically, human wounds heal by forming non-functional fibrotic tissue, commonly referred to as a scar. Among fibroproliferative conditions, keloid and hypertrophic scarring are well-known. These abnormal scars tend to develop in specific areas, such as the anterior chest, shoulder, scapula, and lower abdomen, which are frequently subjected to skin stretching and mechanical forces. Conversely, scars are less common on the scalp and the anterior lower leg due to the direct connection of skin to underlying bones [17]. Moreover, the presence of skin tension lines underscores the significance of anisotropic mechanical properties within the skin’s anatomy. These lines, delineated by the arrangement of dermal collagen and elastin fibers, guide surgeons in making incisions that minimize the likelihood of scar formation [18]. These clinical observations strongly indicate that mechanical forces play a primary role in the formation of scars on wounded skin.

### Laboratory evidence

As research progressed, the molecular and cellular basis for mechanical force-induced scarring is gradually discovered in the laboratory. Mechanosensitive proteins, cells, and biological processes lose their original equilibrium state under the influence of mechanical force and become risk factors for scar formation.

#### Mechanosensitive TGF-β

**Fig. 1:**
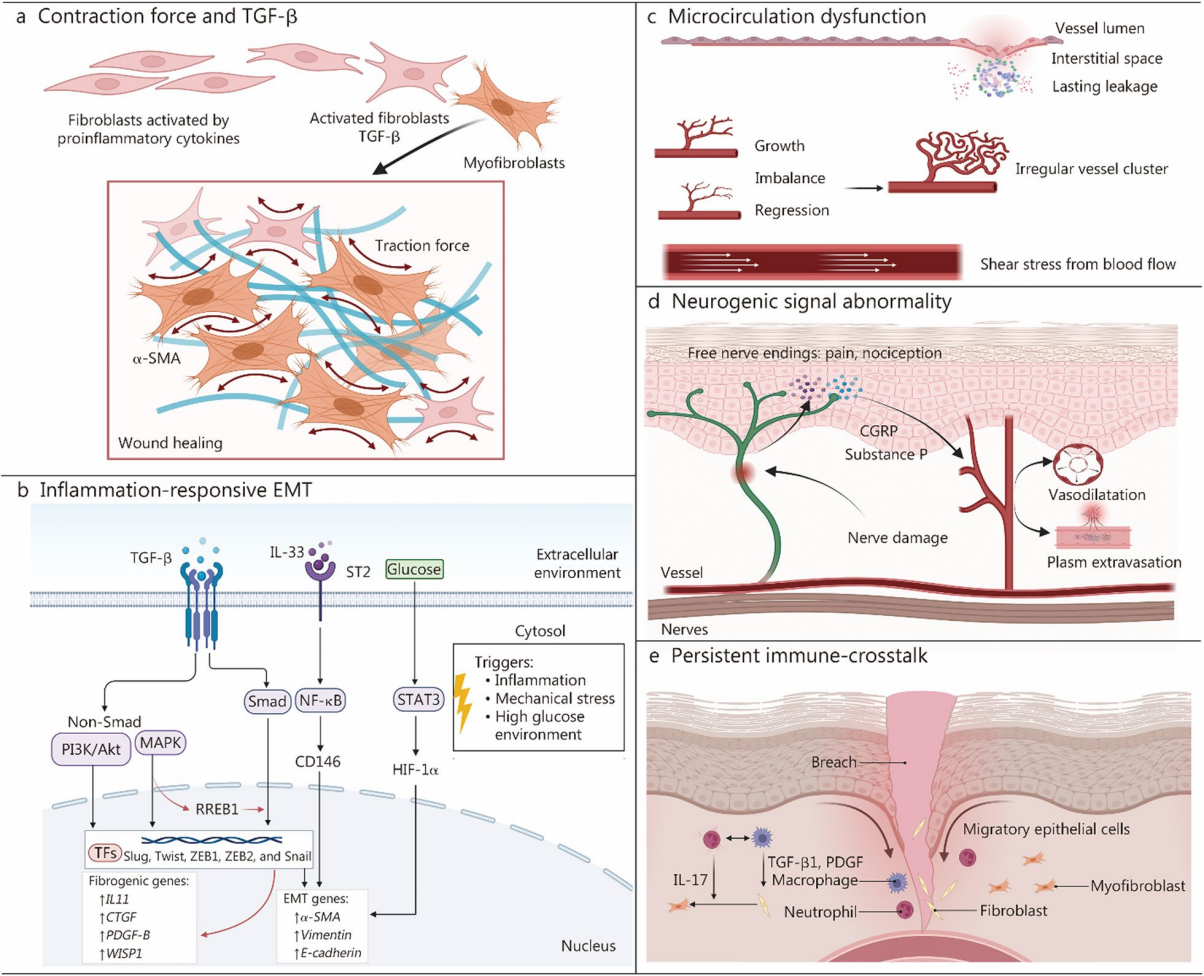
Mechanical cue-dependent scarring. **a** TGF-β activates fibroblasts to transform into myofibroblasts and the latter give rise to α-SMA, endowing wound aberrant contraction force. **b** Epithelial-mesenchymal transition (EMT) in inflammatory environment causes fibrosis related gene expression through diverse pathways. RAS-responsive element binding protein 1 (RREB1) serves as a connector between MAPK and Smad pathways. **c** Inflammatory factors leak from damaged vascular endothelium; imbalance between growth and regression of neovascularization causes irregular vessel cluster; blood flow in irregular vessel produces aberrant shear stress. **d** Nerve damage within wound launches neurogenic inflammation where neuropeptides [calcitonin gene-related peptide (CGRP) and substance P] from nerve endings mediate harmful vascular events including abnormal vasodilation and plasm extravasation. **e** Neutrophiles and macrophages, as the main innate immunocytes within wound, upregulate inflammatory factors and provide fibroblasts with enduring activating signals. Created with BioRender.com. TGF-β transforming growth factor-β, α-SMA α-smooth muscle actin, ST2 suppression of tumorigenicity 2 receptor, PI3K/Akt phosphatidylinositol-3-kinase/Akt, MAPK mitogen-activated protein kinase, TFs transcriptional factors, ZEB1 zinc finger E-box binding homeobox 1, ZEB2 zinc finger E-box binding homeobox 2, IL11 interleukin-11, CTGF connective tissue growth factor, PDGF-B platelet-derived growth factor B, WISP1 Wnt1-inducible signaling pathway protein 1, NF-κB nuclear factor-κB, STAT3 signal transducer and activator or transcription 3, HIF-1α hypoxia-inducible factor-1α

Notably, there is a growing interest in mechanically sensitive proteins, both in in vivo and in vitro studies. Among these proteins, TGF-β stands out as a well-established key cytokine that regulates the response of fibroblasts to injury and the pathological development of fibrosis [19]. Brief mechanical stretching of tissues reduces the levels of soluble TGF-β and collagen I. In contrast, sustained mechanical stimuli increases TGF-β levels, promoting the transformation of fibroblasts into myofibroblasts that express higher levels of α-smooth muscle actin (α-SMA). This transition exacerbates wound contraction force. Moreover, fibroblasts within mechanically loaded wounds undergo a more pronounced fibroproliferative process due to tension, which inhibits the phosphatidylinositol-3-kinase (PI3K)/Akt pathway associated apoptosis [20]. Thus, a detrimental cycle forms within wounded skin where the mechanical force derived from myofibroblasts in turn activate more fibroblasts in a manner of positive feedback to exacerbate scarring [20, 21] (Fig. 1a).

#### Scar-associated cell phenotype

Mechanical forces profoundly influence cell phenotypes [22, 23]. EMT is a pivotal event in wound healing, wherein epithelial cells acquire fibroblast-like features characterized by reduced intercellular adhesion and increased motility. This process is largely stimulated by TGF-β within wounds, resulting in decreased E-cadherin and increased vimentin and α-SMA levels. Injury-induced EMT plays a crucial role in fibrosis processes, including renal, pulmonary, cardiac fibrosis, and cutaneous scar formation due to aberrant TGF-β signaling [24]. Key transcription factors involved in TGF-β-induced EMT include Slug, Twist, zinc finger E-box binding homeobox 1 (ZEB1), ZEB2, and Snail, with signaling pathways encompassing Smad, PI3K/Akt, and mitogen-activated protein kinase (MAPK) pathways [9, 15, 24]. Notably, RAS-responsive element binding protein 1 (RREB1) acts as a bridge between TGF-β-Smad and Ras-MAPK, promoting fibrosis and scar formation by increasing the deposition of fibrous connective tissue, including interleukin (IL)-11, connective tissue growth factor (CTGF), platelet-derived growth factor B (PDGF-B) and WNT-inducible signaling pathway protein 1 (WISP1) [9]. Additionally, IL-33, primarily expressed and stored in the nuclei of endothelial and epithelial cells, contributes to organ fibrosis via type-2 EMT through the IL-33-suppression of tumorigenicity 2 receptor (ST2)-NF-κB p65-CD146 axis [25, 26]. In a high glucose environment, signal transducer and activator of transcription 3 activation increases hypoxia-inducible factor-1α levels and EMT [27] (Fig. 1b). In animal models, insulin, insulin growth factor receptors, protein kinase B, VEGF, and VEGF receptors are upregulated following acute stretching [28]. While these factors enhance vascularity, they also lead to skin microcirculation dysfunction, which is detrimental to scarless wound healing [29]. Various factors contribute to this dysfunction, including high blood vessel permeability, a disrupted neurovascular network, irregular formation of vascular clusters [12] (Fig. 1c), and endothelial mesenchymal transition influenced by shear stress, where endothelial cells acquire a mesenchymal phenotype and express α-SMA and collagen I [30, 31]. Furthermore, the release of neuropeptides (calcitonin gene-related peptide, substance P) by sensory endings is affected by mechanical stress through mechanosensitive nociceptors [10], because nociceptors with unmyelinated axons (C-fibers) are sensitive to compression and tensile. Its pro-scarring essence is nerve-mediated vascular-centric inflammation (Fig. 1d).

#### Mechanical force-related inflammation

In addition to mechanosensitive proteins, immunocytes especially neutrophils and macrophages also respond to mechanical forces and increase the level of inflammatory factors during healing, which is seen as another culprits for fibrosis and scar formation [32] (Fig. 1e). Neutrophils are the first “warrior” migrating from blood to impaired sites, releasing chemokines and cytokines to modulate inflammatory links, killing pathogens via phagocytosis, degranulation, reactive oxygen species, and neutrophil extracellular traps (NETs) [33, 34]. NETs could promote the transition of fibroblasts to myofibroblasts through IL-17 [35]. Normally, by the time of inflammatory phase ending, neutrophils experience apoptosis and are engulfed by macrophages, providing prerequisite for subsequent healing process [36]. However, malapropos behaviors of neutrophils and macrophages contribute to high level of inflammatory factors. Accumulated neutrophils, can be triggered by over-released recruitment promoters such as tumour necrosis factor-α and leukotriene B4 [37], and the dysfunctional phenotype transition of macrophages from pro-inflammatory (M1) to anti-inflammatory (M2) [11]. Macrophage is considered as the main initiator of fibrosis [38]. Indirectly, macrophages produce profibrotic mediators that activate fibroblasts, including TGF-β, PDGF, etc. Directly, matrix metalloproteases and tissue inhibitors of matrix metalloproteinases from macrophages control the turnover of extracellular matrix (ECM). Collectively, overactivation of immune cells under the influence of mechanical forces is one of the important factors in scarring.

## Mechanism of mechano-transduction

**Fig. 2:**
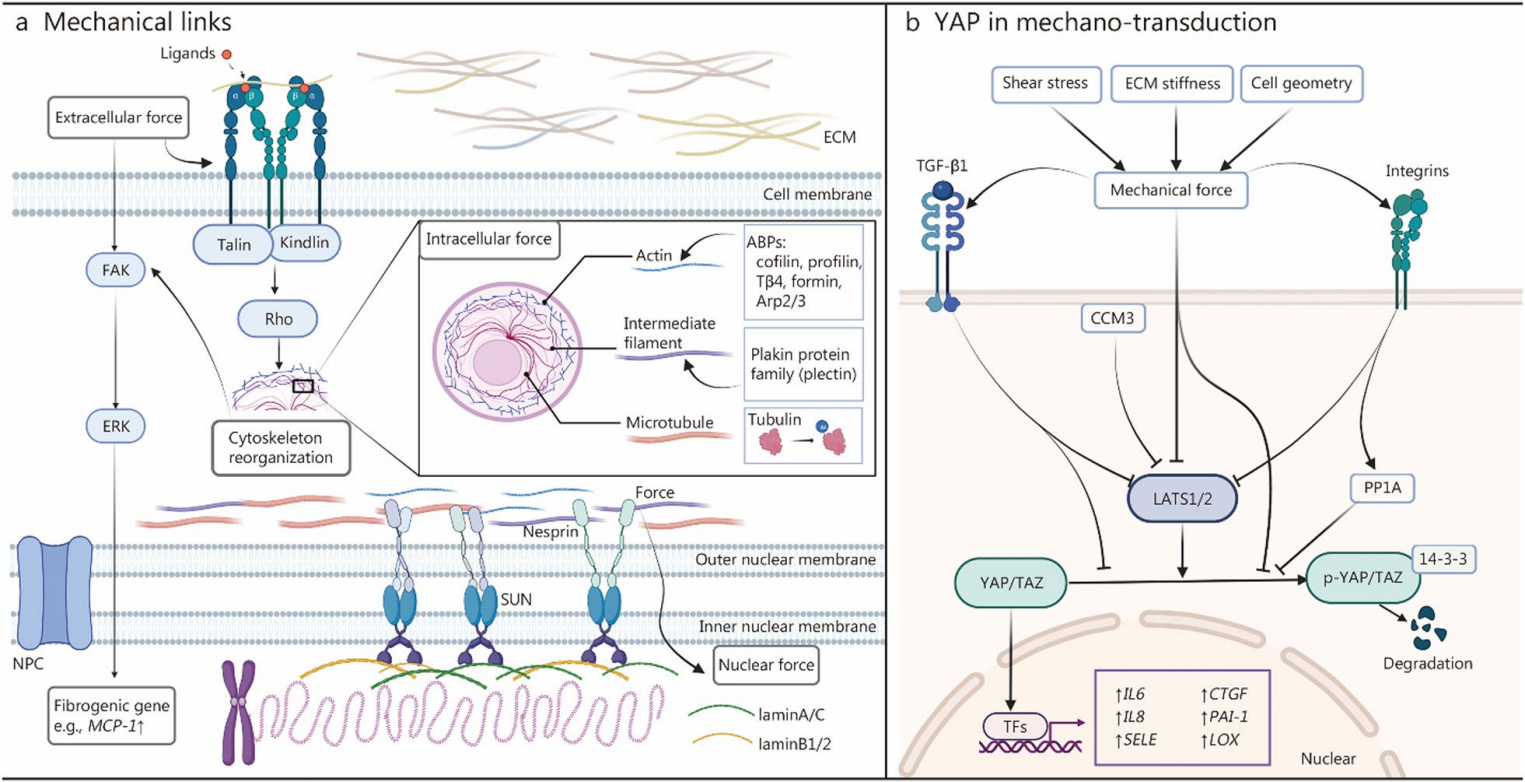
Mechano-transduction and YAP/TAZ. **a** Integrin-cytoskeleton-nesprin-SUN-domain protein serves as a mechanical axis passing mechanical force from extracellular matrix to nuclear lamina. Mechano-sensitive integrin mediates extracellular force induced fibrogenic gene (e.g., *MCP-1*) expression and cytoskeleton reorganization through focal adhesion kinase (FAK)-extracellular regulated protein kinases (ERK) signaling and Rho GTPase, respectively. Intracellular force likewise affects cytoskeletal rearrangement through regulating actin-binding proteins (ABPs), plakin protein family and modification of tubulin (e.g., methylation). Nuclear force regulates chromatin status through interacting with nuclear laminA/C and laminB1/2. **b** Physical factors in extracellular environment (e.g., shear stress, stiffness, cell geometry) produce mechanical forces. These mechanical forces are transmitted by mechanosensitive transmembrane proteins like TGF-β1 and integrins as well as intracellular proteins, like CCM3, to regulate translocation of YAP/TAZ directly and indirectly. YAP/TAZ is directly phosphorylated by LATS1/2 and retains in cytoplasm to combine with protein 14-3-3 and degrade. Mechanical forces inhibit the phosphorylation of YAP/TAZ to increase the translocation of YAP/TAZ into nucleus, which will induce fibrosis related gene (e.g., *IL6, IL8, SELE*) expression. Created with BioRender.com. YAP/TAZ Yes-associated protein and transcriptional coactivator with PDZ-binding motif, FAK focal adhesion kinase, ERK extracellular signal-regulated kinase, NPC nuclear pore complex, MCP-1 monocyte chemoattractant protein-1, Tβ4 thymosin β4, Arp2/3 actin-related protein2/3, ECM extracellular matrix, TGF-β1 transforming growth factor-β1, CCM3 cerebral cavernous malformation 3, LATS1/2 large tumor suppressor 1/2, PP1A protein phosphatase 1α, IL interleukin, SELE selectin E, CTGF connective tissue growth factor, PAI-1 plasminogen activator inhibitor-1, LOX lysyl oxidase, TFs transcriptional factors

Mechano-transduction refers to the process in which cells transmit mechanical forces to intracellular biochemical signals. Mounting studies indicate improper mechano-transduction is an important link in mediating mechanical forces to promote scarring [39]. Cells rely on crucial mechanical components, including focal adhesions (FAs), cytoskeleton, as well as the linker between the nuclear skeleton and cytoskeleton to effectively respond to mechanical forces from sheer stress, matrix stiffness, contraction, etc. (Fig. 2a). This mechano-transduction path is the key to directing biomaterial-based mechanical regulation.

### Integrin-focal adhesion kinase (FAK) signaling

FAs, which connect the actin cytoskeleton to the ECM, consist of transmembrane integrin heterodimers, actin-linking proteins (e.g., vinculin and paxillin), signaling proteins, FAK and so on. The autophosphorylation of FAK plays a crucial role in regulating the assembly and maturation of FAs in response to mechanical forces from ECM perturbations. This process helps maintain local harmony and regulates cell behavior [40]. Specifically, integrin tails bind to cytoplasmic proteins like talin and kindlin, while their extracellular parts bind to ligand proteins in the ECM. When integrin receptors are activated, downstream effectors like Rho GTPases come into play, influencing the reorganization of the cytoskeleton. The expression of integrins is tissue-specific and there are 8 β subunits and 18 α subunits in vertebrates. β1 is expressed in most cell types and it binds collagen, RGD (tripeptide Arg-Gly-Asp sequence), laminin, and leukocyte-specific receptors. Epithelial cells utilize α3 and α6 to interact with laminin, while fibroblasts and endothelial cells rely on α5β3 for cell spreading, and β3 serves as a direct sensor for shear stress [41–43]. In the skin, specialized hemidesmosomes (HDs) anchor keratinocytes to the basal membrane via intermediate filaments (IFs), and α6β4, a transmembrane receptor for laminin-332, is vital for HD formation [44, 45]. Interestingly, integrin α6β4 reduces the size of FAs, cellular spreading, traction force, and interferes with mechano-transduction-related signaling pathways [46]. FAs and HDs appear to have opposite roles, where FAs dominate keratinocyte motility, while HDs guide their settlement. FAs exert significant influence on the biological behavior of various cell types, including differentiation and migration. For example, endothelial cells display distinct cellular morphologies when placed on planar bio-adhesive surfaces or micro-grooved topographic surfaces due to the assembly of FAs [47]. In fibroblasts, mechanical forces transmitted via FAK activate extracellular-related kinase and downstream pro-fibrotic targets, ultimately contributing to the formation of cutaneous scars [48]. Inhibition of FAK in the wound tissue of red duroc pigs resulted in the regeneration of HFs, subcutaneous glands, and peri-follicular adipose tissue [49]. Consequently, targeting the formation of FAs in a mechanical manner holds significant potential to promote scarless wound healing by regulating resident cells within the wound.

### Cytoskeleton and associated proteins

The cytoskeleton consists of three main components: microfilaments, microtubules and IFs. Monomeric G-actin molecules polymerize into actin filaments (F-actin), which are dynamically involved in numerous cellular processes throughout a cell’s lifespan. They interact with a diverse array of actin-binding proteins (ABPs) to influence cellular behaviors [50]. Notably, actin networks, such as lamellipodial and filopodial structures, play pivotal roles in cell polarity during migration and division [51]. The RhoA-Rho kinase/myosin light chain (ROCK/MLC) signaling axis regulates actomyosin contractility and mechanical forces, which is crucial for migration and morphogenesis [52]. Applying forces to actin filaments lead to conformational changes and ABP binding, offering intriguing possibilities for artificial manipulation of cellular behaviors [53]. Profilin, thymosin-β4, formin and the Arp2/3 complex are potential ABPs involved in the mechanical regulation of scarless wound healing [54].

Microtubules exist within the cytoplasm and are highly dynamic. Tubulin and tubule-binding proteins form the hollow cylinder structure of microtubules, serving as the intracellular transport network. Together with accessory proteins, microtubules also play a central role in forming the mitotic spindle, cilia, and flagella [55]. Tubulin modification, such as acetylation, impacts cellular behaviors by promoting the turnover of FAs during cell migration because of mechanical forces from substrate stiffness [56, 57]. Except for the traditional identity as skeletal component which typically supports, maintains the cell shape, microtubule functioning as perceiver of strain is largely coupled with FAs and actin filament [58].

IFs constitute another fibrous component of the cytoskeleton. The molecular composition of IFs varies across cell types. For instance, vimentin is expressed in mesenchymal cells, fibroblasts, and endothelial cells, while keratin is expressed in keratinocytes [59]. Desmin is found in smooth, skeletal, and cardiac muscles [60]. The cell-specific distribution of IFs suggests diverse spatial and temporal biological functions. IFs interact with actin filaments and microtubules, participate in the formation of FAs, and serve as mechano-sensors. For example, keratin IFs in keratinocytes preferentially align on stiff substrates [61]. IFs are anchored to the cell surface by cyto-linker proteins, such as the plakin protein family [62]. Plectin, a member of this family, links keratin filaments and integrin to impact the level of stiffness-dependent laminA/C in keratinocytes [61]. Plectin also plays a vital role in the stable assembly of IFs in fibroblasts by interacting with vimentin [63]. The cytoskeleton’s sensitivity to mechanical cues and its critical role in cellular activities have profound significance for biomaterial design.

### Linker of nucleoskeleton and cytoskeleton (LINC) complex

The LINC complex plays a pivotal role in bridging the connection between the nuclear skeleton and cytoskeleton. Spanning the nuclear envelope, the LINC complex serves as a delivery path for mechanical forces [64]. Its key components include nesprins, which extend across the outer nuclear membrane and interact with cytoskeletal components, and Sad, UNC-84 domain protein (SUN), which span the inner nuclear membrane, connecting nesprins to the nuclear lamina [65]. The LINC complex influences gene expression through interacting with perinuclear constituents [66]. Changes in tension within nesprins mechanically modulate cell polarity through β-catenin signaling during EMT [67]. These changes result from actomyosin-dependent perturbations, providing strong evidence of mechanical regulation of the LINC complex [68]. Targeted strategies have been explored to manipulate the LINC complex. For example, the progression of laminA-associated dilated cardiomyopathy involving laminA can be slowed down by disrupting the LINC complex using adenovirus associated virus-mediated delivery of DN-SUN1 to cardiomyocytes [69]. Notably, in epithelial cells, tension generated by integrins around keratinocytes, which then passes through the LINC complex, can be transmitted to the nuclear lamina. This transmission helps maintain the proliferative potential of basal keratinocytes and suggests an adjustable mechanical cue to enhance re-epithelialization in wound healing [70]. In addition, keratin 15, a component of the LINC complex, is found to play a significant role in wound repair by interacting with nuclear constituents [71]. In summary, mechanical links offer considerable potential for mechanical tuning in wound-resident cells.

### Yes-associated protein (YAP) and transcriptional coactivator with PDZ-binding motif (TAZ)

The Hippo pathway and its downstream effectors, YAP and TAZ, emerge as key players in mechano-transduction (Fig. 2b) [72]. In the cytoplasm, the nuclear Dbf-2-related (NDR) family of kinases, primarily large tumor suppressor 1/2 (LATS1/2), directly regulate the phosphorylation of YAP/TAZ. Mechanical cues and signals transmitted through integrins inhibit the phosphorylation of YAP/TAZ, either directly or by inactivating LATS1/2 or activating protein phosphatase 1α. Phosphorylated YAP/TAZ (p-YAP/TAZ) in the cytoplasm binds 14-3-3 proteins, and is subsequently degraded by proteasomes. Hypo-phosphorylated YAP/TAZ enters the nucleus to regulate transcriptional factors, primarily the transcriptional enhanced associate domain family [73, 74]. YAP/TAZ target genes involved in fibrogenesis, such as CTGF, plasminogen activator inhibitor 1 (PAI-1), and the lysyl oxidase (LOX) family of collagen crosslinking enzymes [73]. In dermal fibroblasts, YAP1 can be activated by TGF-β1 and acts as a mediator driving the transition of fibroblasts into myofibroblasts [75]. Fibrogenesis mediated by YAP is also explored in lung and kidney [76, 77]. In the context of cutaneous lesions, YAP/TAZ shuttles between the cytoplasm and nucleus before or after engaging in different signaling cascades, exhibiting complex roles. ECM stiffness and cell geometry regulate the subcellular localization of YAP in mammary epithelial cells through Rho GTPases and cytoskeletal tension [78]. Blood flow, which exerts shear stress on endothelial cells, inhibits YAP/TAZ activity, thereby suppressing pro-inflammatory gene (*IL6*, *IL8*, and *selectin E*) expression related to JNK signaling [43]. Hierarchical regulation exists between FAs and YAP [79]. Study of anisotropic forces suggests that YAP translocation occurs after stretch parallel to the long axis of elongated cells but not perpendicular to the stretch [80]. In mouse skin, YAP activity may activate plasminogen activator, urokinase (PLAU) and TGF-β receptor III to promote keratinocyte proliferation [81]. YAP-mediated mechano-transduction also holds the potential for immune modulation [82]. In postnatal wound healing, regulated by mechanical tension and YAP signaling, Engrailed-1 lineage-negative fibroblasts transform into Engrailed-1 lineage-positive fibroblasts, which are known to play a role in scarring [74]. Therefore, modulating YAP signaling can facilitate scarless wound healing, which indicates a direction for investigating the mechanical interactions between biomaterials and cells.

### Inspiration from mechano-transduction for biomaterial design

Biomaterial stiffness can orchestrate ordered tension, guiding the speed and direction of cell migration reliant on the expansion and contraction of mechanosensitive cytoskeletal elements. Varied shear forces, produced by liquids flowing over biomaterial surfaces of differing roughness, can regulate the fate of circulating blood cells, including vascular endothelial cells, monocytes, and macrophages. These cells naturally experience shear forces physiologically. Deformable biomaterials have the capacity to generate tensile forces, impacting traction-sensitive tissues and organs like neuronal axons and dermal tissues. The adaptability and plasticity of these biomaterials allow for the construction of an appropriate mechanical environment, influencing cell behaviors effectively. The ability to finely tune the mechanical properties of biomaterials offers a promising avenue for controlling and manipulating cell fates during wound healing, providing a framework to optimize tissue repair and regeneration.

## Bioactivities of biomaterials due to biophysical characteristics

**Fig. 3:**
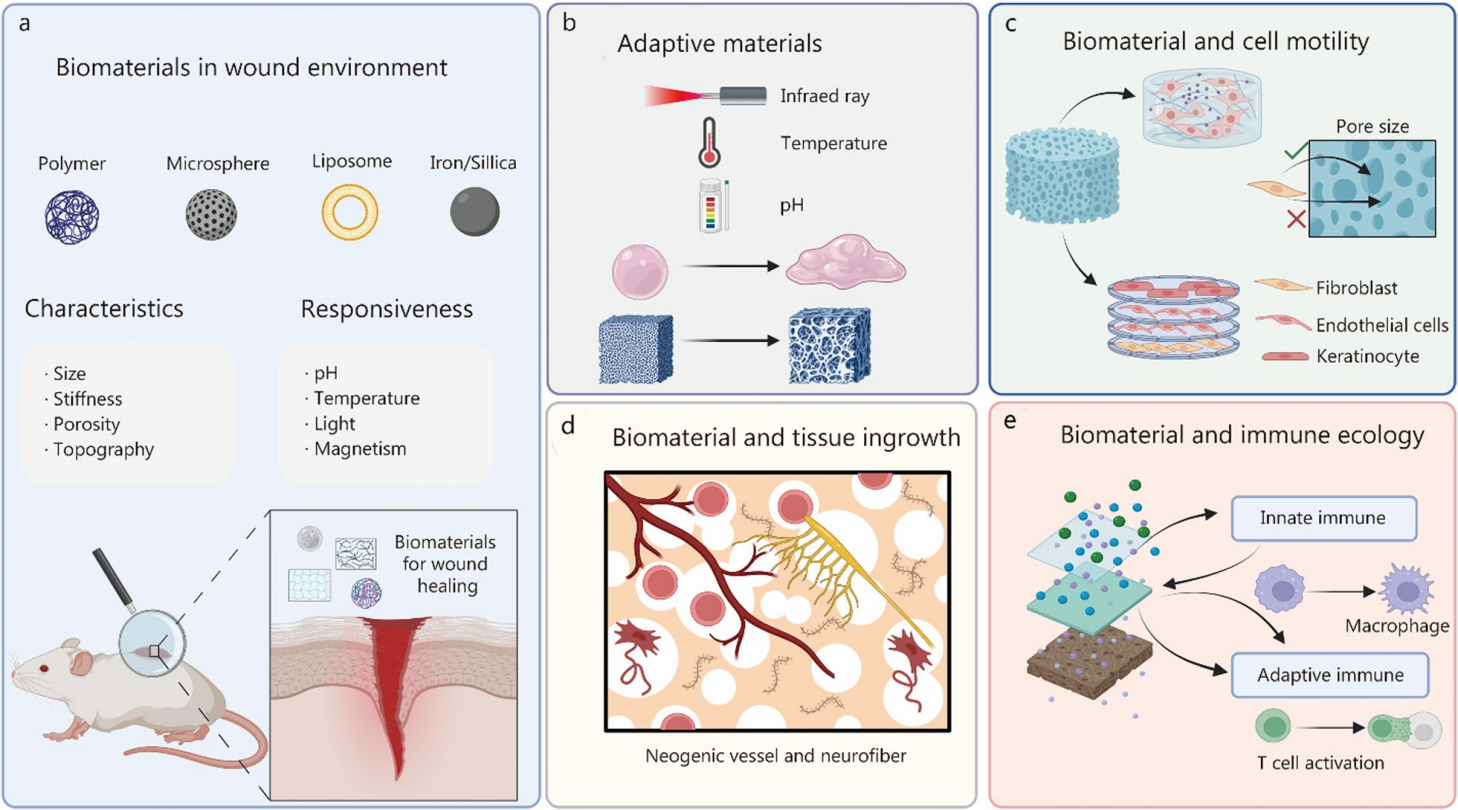
Biomaterials mechanically interact with wound environment. **a** A wide variety of biomaterials (e.g., polymer, mesoporous, liposome, iron/silica) can be designed for wound healing. These materials actively or passively match wound characteristics to provide optimal condition for wound healing. **b** Adaptive materials: adaptive materials actively respond to internal or external stimuli (e.g., pH, temperature, light and magnetism) to change their characteristics (e.g., size, stiffness, porosity, topography) or degrade. **c** Biomaterials for cell motility: engineered biomaterials have the potential to enhance the motility of cells by offering suitable motility speed and direction within a three-dimensional (3D) movement environment. Creating different pore sizes within materials is an important means to achieve multi-dimensional cell movement. **d** Biomaterials for tissue ingrowth: precisely designing the degradation modes and 3D structure of biomaterials plays a pivotal role in allowing tissue ingrowth. Neogenic vessel and neurofiber could gradually grow as materials degrade. **e** Biomaterials for immune ecology: biomaterial surface, topography, wettability, and stiffness regulate phenotype of macrophages, neutrophils (innate immune) as well as T cell activation (adaptive immune). Created with BioRender.com

Biomaterials, owing to their physical properties such as shape, stiffness, elasticity, topography, and static strength (which endows biomaterials with the ability to withstand tension, shear stress, compression, etc.), play a crucial role in regulating mechanical signals during their interaction with cells and the wound environment (Fig. 3a, b). These biomaterials are able to influence cell plasticity through regulating cell adhesion, cytoskeleton, nuclear state, as well as epigenetic modifications, which offers new insights into the fate transformation of wound-resident cells. Furthermore, biomaterials influence the speed and direction of cell motility and regulate tissue ingrowth. Their responsiveness to both biomechanical and biochemical cues allow them to adapt to the wound healing process. Importantly, the physical properties of biomaterials are tunable through various methods, making it feasible to develop biomaterial-based strategies to facilitate wound healing by mechanical means. Bioactivities of biomaterials are summarized in Table 1 [6, 7, 47, 57, 78, 83–101].

**Table 1:** Bioactivities of biomaterials with specific physical property

Cell type	Material	Physical property	Biological function	References
HUVECs	β-TCP	Multiple channels	Directing migration	[6]
HDFs	PHBV	Electro-spun meshes	Decreasing differentiation of fibroblasts to myofibroblasts	[7]
Bovine ECs	PDMS	Micropattern microgroove	Enhancing ECs alignment	[47]
Rat astrocytes	PA	Stiffness; 1.26–48 kPa	Increasing YAP nuclear localization	[57]
MECs	Acrylamide	Stiffness; 0.7–40 kPa	Increasing YAP/TAZ activity	[78]
hES-MP	Poly-HIPE	Porosity	Enhanced adhesion	[83]
hMSCs	PLLA, PS	Nanopatterned surface	Increased focal adhesions	[84]
Mouse MSCs	PDMS	Micropatterned surface	Orderly, quicker, and greater FAs formation	[85]
hFOB	PLLA	Nanoscale pit textures; 14–45 nm deep pits	Different focal adhesions	[86]
Osteoblast	HA	Nanoscale; 10–100 nm	DNA methylation	[87]
hESCs	NSq50 substrate	Nanopit, nanotube	DNA methylation; mesenchymal differentiation	[88]
Cardiac progenitors	Silicon wafers	Microgrooves;10 µm wide, 3 µm deep	Histone H3 acetylation	[89]
Platelet	PA gels	Stiffness; 50–100 kPa	Increasing activation of platelet	[90]
HUVECs	PDMS and PDL	Stiffness; 2.6 Pa and 5 kPa	Supporting migration	[91]
HDFs	C/P/ZnO films	Porosity	Supporting migration	[92]
HUVECs	SiO_2_	Porosity	Supporting migration	[93]
Human LECs	PLLA films	Aligned electro-spun fibers	Tissue ingrowth	[94]
Sciatic neuron	CAB	Nano-grooved fibers	Instructing the size of myelin sheaths and axons	[95]
Macrophages	PA gels	Stiffness; 280 kPa–70 GPa	Supporting migration	[96]
Macrophages	Collagen I and GAGs	Stiffness; (118.5 ± 34) Pa	Instructing polarization	[97]
Mouse MSCs	Alg-Gel blends	Stiffness; 47.5–352.5 kPa	Increasing the differentiation of MSCs to SGCs	[98]
Chondrocytes	GelMA	Stiffness; 3.8, 17.1, and 29.9 kPa	Maintaining chondrogenic phenotype	[99]
HUVECs	GelMA	Elastic modulus of 3.3–110 kPa	Supporting migration	[100]
Schwann cells	PCL	Anisotropic topographies	Nerve growth	[101]

*HUVECs* human umbilical vein endothelial cells, *HDFs* human dermal fibroblasts, *ECs* endothelial cells, *MECs* mammary epithelial cells, *hES-MP* human embryonic stem cell-derived mesenchymal progenitor cells, *hMSCs* human mesenchymal stem cells, *MSCs* mesenchymal stem cells, *hFOB* human fetal osteoblastic cell, *hESCs* human embryonic stem cells, *LECs* lymphatic endothelial cells, *β-TCP* β‑tricalcium phosphate, *PHBV* poly(3-hydroxybutyrate-co-3-hydroxyvalerate), *PDMS* polydimethylsiloxane, *PA* polyacrylamide, *Poly-HIPE* polymerized high internal phase emulsion, *PLLA* poly(L-lactic), *HA* hyaluronic acid, *PDL* poly-D-lysine, *C/P* chitosan/pectin, *CAB* cellulose acetate butyrates, *GAG* glycosaminoglycan, *Alg-Gel* alginate/gelatin, *GelMA* gelatin methacryloyl, *PCL* polycaprolactone, *YAP* yes-association protein, *TAZ* transcriptional coactivator with PDZ-binding motif, *FA* focal adhesion

### Modulating extracellular signaling

Biomaterials modulate extracellular signaling to profoundly impact cellular activities, which suggests the potential for in situ mechanical reprogramming [102]. It is increasingly recognized that optimal cell adhesion is strongly influenced by the physical characteristics of biomaterial surfaces, including electrical charge, topography, and roughness [103, 104]. Key molecules from the extracellular or provisional matrix, such as fibronectin, vitronectin, collagen, laminin, or fibrin, play crucial roles in mediating cell adhesion to artificial biomaterials [105]. Whether biomaterials are employed in vivo or in vitro settings, these extracellular components are naturally drawn to the biomaterials from culture media, blood, and other bodily fluids, or they are deposited by resident cells. This process is essential for cells to establish FAs by binding integrins. Generally, positively charged surface is more conducive to the formation of adhesion because adhesion-mediating ECM molecules are usually negatively charged. While, different cell types have different responses to surface morphology created by various biomaterials. Distinct spatial dimensions affect the maturation of FAs. When cultured on polymerized high internal phase emulsion foams with closed [two-dimensional (2D)] or open [three-dimensional (3D)] pore morphologies, human embryonic stem cell-derived mesenchymal progenitor cells (hES-MP) display enhanced adhesion and cell viability on those with open pores because this open pore structure provides 3D attachment which is better for cells to exert comprehensively interaction [83]. When in the same spatial dimension, anisotropic surfaces prepared through diverse techniques, e.g., lithographic, microfabrication, electrospinning, can induce cell reorientation following microgrooves or aligned bumps and indentations, the so-called contact guidance phenomenon [84]. Micropatterned or planar surface of fibronectin-coated polydimethylsiloxane (PDMS) substrate influence behaviors of C3H10T1/2 cells (murine mesenchymal stem cells). Clear differences between the FA formation and maturation patterns are observed: cells on micropatterned surface displayed more orderly, quicker, and greater FA formation [85]. Therefore, tissue regeneration with specific structure can be directed by specific patterned surface. Nanoscale structure seems to be superior to microscale because basement membranes of various tissues are composed of a complex mixture of nano size (5–200 nm) pits, pores, protrusions, and fibers [86]. Therefore, more diverse cell-specific effects occur on nanoscale surfaces, which is more resemble to physiological conditions. For human mesenchymal stem cells, surface nanopatterning controls the initial assembly of FAs [84], while primary human fibroblasts on nanoscale roughness surface display reduced formation of FA complexes, less spread and an elongated morphology [86]. Still, these biomaterial-based strategies can only provide static surface which are different from real dynamic conditions within organisms. Recent technology has pushed the limits of static control by using shape memory polymers (SMPs). Human cardiomyocytes display different formation of FAs on SMPs coated with polyelectrolyte multilayer which has achieved dynamic nano-wrinkle formation. Specifically, FA proteins like zyxin, vinculin, paxillin align along the wrinkle direction [106]. In addition to inducing novel formation of FAs, biomaterials can also induce FAs sliding thus influencing cell polarization [107]. All of the above are materials that come into contact with cells and thus affect the formation of FAs, non-contact method is developed to exert external mechanical forces through using microfabricated arrays of magnetic and non-magnetic silicone elastomeric posts, and as a result, enhanced FA formation is observed [107]. Biomaterial-based controllable formation of FAs dictates restoration of specific cellular functions. The effect of microenvironment stiffness on the cytoskeleton has been extensively studied. Primary human aortic smooth muscle cells within 3D poly(ethyleneglycol)-fibrinogen hydrogels increase their assembly of F-actin stress fibers and FAs when rigidity increases [104]. Chondrocytes on silicon-based elastomer PDMS substrates with different substrate stiffnesses show changes of secreted extracellular linkage protein, laminin β1, FA complex protein, FAK as well as cytoskeleton reorganization [108]. Evidences show that substrate stiffness mediates the formation of novel cytoskeletal structure within mouse embryonic fibroblast 3T3 cells and rat Schwann cells [109, 110]. Accelerated osteoclast differentiation through cytoskeleton rearrangement is observed on stiffer PDMS substrates which is firstly manifested in variations of their morphology and fusion/fission activities, then in the upregulation of canonical osteoclast markers as well as the activation of cytoskeleton-associated adhesion molecules, including fibronectin and integrin αvβ3, followed by biochemical signaling cascades of paxillin, FAK, and RhoA [111]. It seems that the stiffness that best suits the function of a cell often matches the stiffness of its internal environment. Collectively, the physical properties of biomaterials affect extracellular signaling through regulating cell adhesion.

### Influencing cellular epigenetics

Leveraging mechanical perturbations, biomaterials hold promise as a strategy to overcome epigenetic barriers and control cell fate because mechanical signals from biomaterials are transmitted from the cell membrane to the nucleus, directly influencing chromatin structure and gene expression [112]. Epigenetics encompass heritable alterations in gene function that occur without modifying the DNA sequence, ultimately resulting in observable changes in an organism’s traits or characteristics. The epigenome plays a crucial role in maintaining the stability of cell identity, especially in situations such as stem cell differentiation and somatic cell reprogramming, where cellular states can become disrupted. Epigenetic regulation involves common mechanisms such as chromatin remodeling, DNA methylation, and histone protein modifications. For instance, the stiffness of hydrogels plays a key role in reprogramming mouse embryonic fibroblasts into induced pluripotent stem cells through mesenchymal-to-epithelial transition and the regulation of stemness markers. Stiff surfaces promote an open and transcriptionally active euchromatin structure, while soft biomaterials induce a dense and transcriptionally inactive heterochromatin structure [23]. Nanomaterials, like nano-hydroxyapatite, have the capability to influence osteoblast lineage commitment by promoting DNA methylation and regulating the expression of key osteoblastic marker genes [87]. When human embryonic stem cells are cultured on nano-topographical substrates, they exhibit increased expression of mesenchymal and stromal markers, along with elevated levels of early osteogenic progenitors, compared to cells cultured on flat substrates in the same basic media conditions. Additionally, during substrate-induced differentiation, these cells undergo epithelial-to-mesenchymal transition, and they display DNA methylation changes akin to those induced by chemical factors [88]. Micro-grooved substrates enhanced the directed differentiation of cardiac progenitors into cardiomyocyte-like cells through promoting histone H3 acetylation [89]. Increasing accessibility of DNA and epigenetic modification is the prerequisite for cell fate turnover.

### Supporting cell migration

Engineered biomaterials have the potential to enhance the flexible motility of cells by offering suitable motility speed and direction within a 3D movement environment [113] (Fig. 3c). Cells are inclined to migrate towards regions that match their optimal stiffness, where they can generate maximal traction [114]. Skin rupture disrupts the harmonious cellular community. Wound-resident cells, including epidermal stem cells, fibroblasts, and endothelial cells, mobilize collaboratively to facilitate wound closure. It’s noteworthy that platelet spreading and activation intensify when adhered to fibrinogen-coated polyacrylamide (PA) gels with greater stiffness [90]. Endothelial cells are more active on stiffer PDMS [115]. Keratinocyte cytoskeleton adapts to matrix rigidity on PA hydrogels [61]. Fibroblasts influenced by stiffness may lead to over-recruitment and fibrosis [116]. Hence, materials featuring stiffness gradients hold the potential to guide the migration of wound-resident cells and enhance their motility, ultimately improving the efficacy of wound healing. Numerous techniques can be employed to create materials with stiffness gradients. One approach involves segmentally controlling the proximity of reactants to regulate the degree of crosslinking. To illustrate, by leveraging differential diffusion distances of unreacted cross-linkers and monomers within a pre-polymerized hydrogel matrix, PA hydrogels were successfully engineered. These hydrogels exhibit linear stiffness gradients ranging from 0.5 to 8.2 kPa/mm, covering the mechanical spectrum observed in both physiological and pathological in vivo conditions [117]. Moreover, another effective approach involves overlaying films with varying moduli. For instance, one can position a thin, low modulus PDMS film, atop a high modulus PDMS structure. This technique not only allows the creation of flat surfaces with distinct stiffness patterns but also provides a means to introduce surface undulations [118]. Detecting micro-level stiffness is challenging, prompting the application of computational modeling based on filopodia mechano-sensing to predict and understand directed cell migration [91]. While proper stiffness facilitates flexible 2D cell motility, porosity is essential for enabling cells to infiltrate various anatomical layers, thereby offering a more realistic stereoscopic environment. Engineered porous biomaterials are designed to segregate different cell types and govern their interactions. By controlling the size of the pores, for instance, one can confine fibroblast activity to the dermal layer, allowing precise control over cellular behavior at distinct anatomical levels. A recent innovation involves the development of 3D porous films composed of chitosan, pectin, and ZnO, featuring pore sizes ranging from 99 to 126 nm. These films have been designed to expedite scarless wound healing [92]. Porous substrates promote the migration of endothelial cells, thereby facilitating and enhancing the process of wound healing [93]. Engineered porous β-tricalcium phosphate scaffolds have been utilized to address bone defects by stimulating cell migration, proliferation, and angiogenesis. These scaffolds serve as potential supporters of wound-resident cells [6]. Therefore, biomaterial-based biomimetic scaffolds are interesting to regulate cell motility.

### Allowing tissue ingrowth

Precisely designing the degradation modes, 3D structure, and electrical properties of biomaterials plays a pivotal role in allowing tissue ingrowth [119] (Fig. 3d). Timely degradation of these materials is crucial to avoid unnecessary clinical interventions which is detrimental to developing tissue. As biomaterials degrade into smaller components, they not only create space for tissue growth but also enable the controlled release of trophic factors, including growth factors, cytokines within specific spatial and temporal constraints of the wound site. Several factors influence the degradation of biomaterials, including environmental factors such as temperature, pH, and fluid dynamics, as well as material attributes like porosity, surface patterns, and shape. The mode of degradation varies depending on the type of materials used. Materials like polylactide, polyglycolide, and polycaprolactone rely on hydrolysis, but their degradation rates differ, and direct contact with water molecules significantly affects the degradation process. Consequently, during the early phase of wound healing, when there is a substantial amount of fluid effusion, the degradation of hydrolytically unstable materials can be accelerated [120]. Premature degradation compromises the biomaterials’ ability to serve as a barrier against external harmful factors. As the proliferative phase begins, it’s imperative that biomaterials do not occupy excessive space, as more room is needed for tissue ingrowth. Hence, creating a 3D environment through sequential degradation for flexible cell movement serves as the foundation for tissue growth. Zhao et al. [121] and Xu et al. [122] have focused on developing a silicone rubber membrane (SRM). They initially crafted a single-layer SRM with an adjustable microporous structure, achieved through solvent evaporation-induced phase separation. Then, they constructed a silicone rubber membrane bilayer (SRM-B) consisting of upper and lower layers differing in thickness, pore size, and pore density. The smaller pores serve to prevent bacterial infection, while the larger ones promote cell adhesion and proliferation, thereby expediting re-epithelialization [121, 122]. Nanoscale biomaterials are easier to degrade and be absorbed because of the small size effect, which provides a higher specific surface area. The nanoscale dimensions make biomaterials more adept at mimicking the in vivo physical environment, thereby enhancing their effectiveness in therapeutic applications [5, 123–125]. In recent years, the electrical properties of biomaterials have gained attention as a promising parameter to replicate native biological electric activity. The concept of an enzymatic biofuel cell was initially introduced in 2015 as a means to serve as an electrical reservoir, generating electric stimuli to support regeneration and repair processes. By employing enzymes like glucose oxidase and bilirubin oxidase, enzymatic biofuel cells have demonstrated remarkable performance in muscle tissue regeneration, facilitating cell proliferation, differentiation, and migration [126]. A similar device has been applied to create a wound dressing, which combines a porous PA hydrogel, referred to as an “electricity auto-generating glucose-responsive enzymatic-biofuel-cell skin patch”. When this innovative patch is employed on wounded rat skin, it significantly improves the efficiency of wound closure, neovascularization and matrix deposition, demonstrating its potential as a valuable tool for wound healing [127]. While cells exhibit specific behaviors, achieving tissue ingrowth necessitates the establishment of connections and orderly arrangements. A comprehensive understanding of skin anatomy through tomography serves as inspiration for designing biomaterials with stereoscopic structures, ensuring adequate space for the growth of various tissues. The exploration of multifunctional composite biomaterials that facilitate tissue growth should be a priority for further research and development.

### Adapting to wound environment

Technological advancements have shown that biomaterials with favorable physical properties can be tailored through various methods to adjust and adapt to the wound environment, thereby optimizing the conditions for regenerative healing (Fig. 3b). When breaches occur, the continuity of the skin is disrupted, leading to physical and chemical changes such as unevenly distributed mechanical tension, alterations in electric fields due to injury-induced ion redistribution, shifts in temperature, pH levels, and metabolite concentrations. Hydrogels have consistently shone in the field of wound healing. They can be derived from various sources, including gelatin, collagen, fibrin, hyaluronic acid, chondroitin sulfate, alginate, chitosan, poly(ethylene glycol), and poly(vinyl alcohol) [128]. While hydrogels share similarities with the ECM, their inherent lack of stiffness limits their ability to withstand tension and support tissue ingrowth effectively. To address this challenge, researchers focus on modifying gelatin hydrogel, creating variants with diverse mechanical properties using various methods. One approach involves directly reacting gelatin with methacrylic anhydride, resulting in the synthesis of gelatin methacryloyl (GelMA). The degree of methacrylation plays a crucial role in determining the elastic modulus of GelMA. Additionally, by incorporating self-assembled chitin nanofibers, micropatterned GelMA hydrogels with enhanced stiffness and strain properties have been developed. These modified hydrogels show the capacity to induce cell differentiation and vasculo-genesis, simplifying their utility in regenerative applications [100]. Self-adaptivity of biomaterials can be triggered by factors such as temperature, pH, metabolite concentrations, or external stimuli like light of varying wavelengths, magnetism, and electrical stimulation. In particular, fibrous GelMA scaffolds show superior performance compared with GelMA hydrogels. This is attributed to their ECM-like nanofibrous structure, which provides a higher surface-to-volume ratio, facilitating closer interaction between the materials and the wound environment. To simplify the process, researchers combined radical photo-crosslinking with reactive methacryloyl groups. This approach achieves the temporal and spatial control of the physical properties of GelMA hydrogel, including water retention, stiffness, strength, elasticity, and degradation, simply by altering the exposure time to light [129]. Contact-free methods for handling materials are preferred to avoid disrupting the healing process. In addition, temperature plays a crucial role in wound healing. Considering the temperature difference between deep wounds (37 °C) and skin surface (25 °C), a hydrogel with dual characteristics of fluid-like and solid-like properties has been developed. This innovative hydrogel is designed to provide a soft setting in deep wounds, while also serving as a robust barrier for irregularly shaped skin wounds. The inspiration for this ingenious self-adaptive hydrogel is drawn from the temperature-dependent viscoelasticity of polymers formed by borate ester bonds [130]. The pH value undergoes frequent changes during the healing process, which can be detrimental to biological reactions. Healthy skin typically maintains a pH range of 4–6. In contrast, wounds with pus, necrotic tissue, and serum crusts often exhibit a mean pH value of around 6.1, while chronic or infected wounds have a pH of approximately 7–8. Interestingly, wound debridement appears to lead to an increase in pH levels [131]. A pH range of 4–6 is considered conducive to effective wound healing, and pH-sensitive biomaterials can be employed to maintain an optimal pH environment within wound. In the case of protein-based hydrogels, their pH sensitivity is attributed to the presence of ionizable amino acids. Elastin-like polypeptides (ELPs) can be designed to incorporate ionizable amino acids like lysine, tyrosine, and valine. These ELPs can then be crosslinked to create hydrogels with adjustable pH sensitivity through chemical modification [132]. In order to monitor pH fluctuation within wound, pH-sensitive PDMS optical fibers are integrated into carborxymethy chitosan-protocatechualdehyde (PA)@Fe hydrogels to create a smart visible monitor system [133]. Furthermore, in the case of chronic wounds, particularly those associated with diabetes, elevated sugar levels can be leveraged in the design of responsive biomaterials. This responsiveness is achieved through the competitive binding of sugar to poly(amidoamine) dendrimers bearing phenylboronic acid (PBA-PAMAM). Notably, layer-by-layer thin films that incorporate PBA-PAMAM can degrade in response to changes in sugar levels [134]. Moreover, responsive biomaterials can enhance advanced strategies for wound healing. For instance, somatic cells undergoing in situ differentiation into pluripotent cells endow wounded skin with remarkable regenerative potential. However, during the process of somatic cell reprogramming to pluripotent cells, several changes occur, including shifts in extracellular pH from alkaline to acidic, alterations in metabolic pathways from oxidative phosphorylation to glycolysis, and rearrangement of the cytoskeleton. To harness these changes, a cell reprogramming-responsive hydrogel is developed. This hydrogel is designed to facilitate the expansion of cell clusters by adjusting its mechanical properties in response to the aforementioned alterations, thereby promoting the transformation of cell fate and enhancing regenerative healing potential [135]. Biomaterials can likewise be engineered to be responsive to various factors associated with scarring such as EMT, contraction forces, and fibroblast activation. These responsive biomaterials can play a pivotal role in regulating collagen deposition, alleviating mechanical tension, and influencing the behavior of fibroblasts, ultimately contributing to improved wound healing outcomes by orchestrating these critical cellular and mechanical processes.

## Potential of biomaterial-based mechanical regulation for skin appendage regeneration

Mechanical regulation has been demonstrated effective in tissue development and regeneration [136]. On the one hand, biomaterial-based mechanical regulation is expected to provide a scar-free environment which is an important prerequisite to regenerating skin appendages. On the other hand, biomaterials regulate cellular plasticity through mechanical means which will be an effective way to expand the precursor cell bank. Biomaterials for skin appendage regeneration are discussed below.

### Scarless environment: a prerequisite for skin appendage regeneration

Post-injury tissue fibrosis repair often results in a reduction of functional tissue structure and cell diversity, accompanied by an increase in fibrous tissue formation. This shift can be detrimental to the restoration or regeneration of skin appendages [137]. Therefore, the improvement of the fibrotic environment is essential for successful appendage regeneration. From the perspective of embryogenesis, the formation of skin appendages depends on the crosstalk of the dermis with the epidermis. Specifically, scattered cells within the epidermal plane produce a higher WNT signal than their neighbors, and these cells cluster into placodes [138, 139]. If the underlying mesenchyme produces elevated levels of bone morphogenetic protein (BMP) inhibitors, the WNT^high^ cells will form an HF [140]. If the underlying mesenchyme produces a strong BMP signal, the WNT^high^ cells will make a SwG [137]. Postnatally, close intercellular interactions between epithelial and dermal cells maintain and control the hair growth. Signals or soluble factors from dermal cells in hair bulbs directly influence the adjacent epithelial cells [141, 142]. However, in the context of scarring, dermal fibroblasts which are the main producer of mesenchyme signals are activated by various stimuli to experience phenotypic transformation to myofibroblasts. This transformation rapidly creates a pro-fibrotic environment within wound in a cascading manner, thus completely destroying the mesenchymal environment in which appendages grow [143–145]. Therefore, we highly confirm the necessity of biomaterial-based scarless wound healing. Bioactivities of biomaterials with specific physical properties make biomaterials a promising avenue for preventing scarring by mechanical means. Specifically, elastomeric materials can be employed to regulate collagen deposition and biomaterials with specific surface patterns and topographies can facilitate functional neovascularization and the restoration of innervation. Given the diverse biological processes involved, biomaterials often need to be tailored to specific requirements and these can be translated into quantifiable parameters.

#### Elastomeric materials

**Fig. 4:**
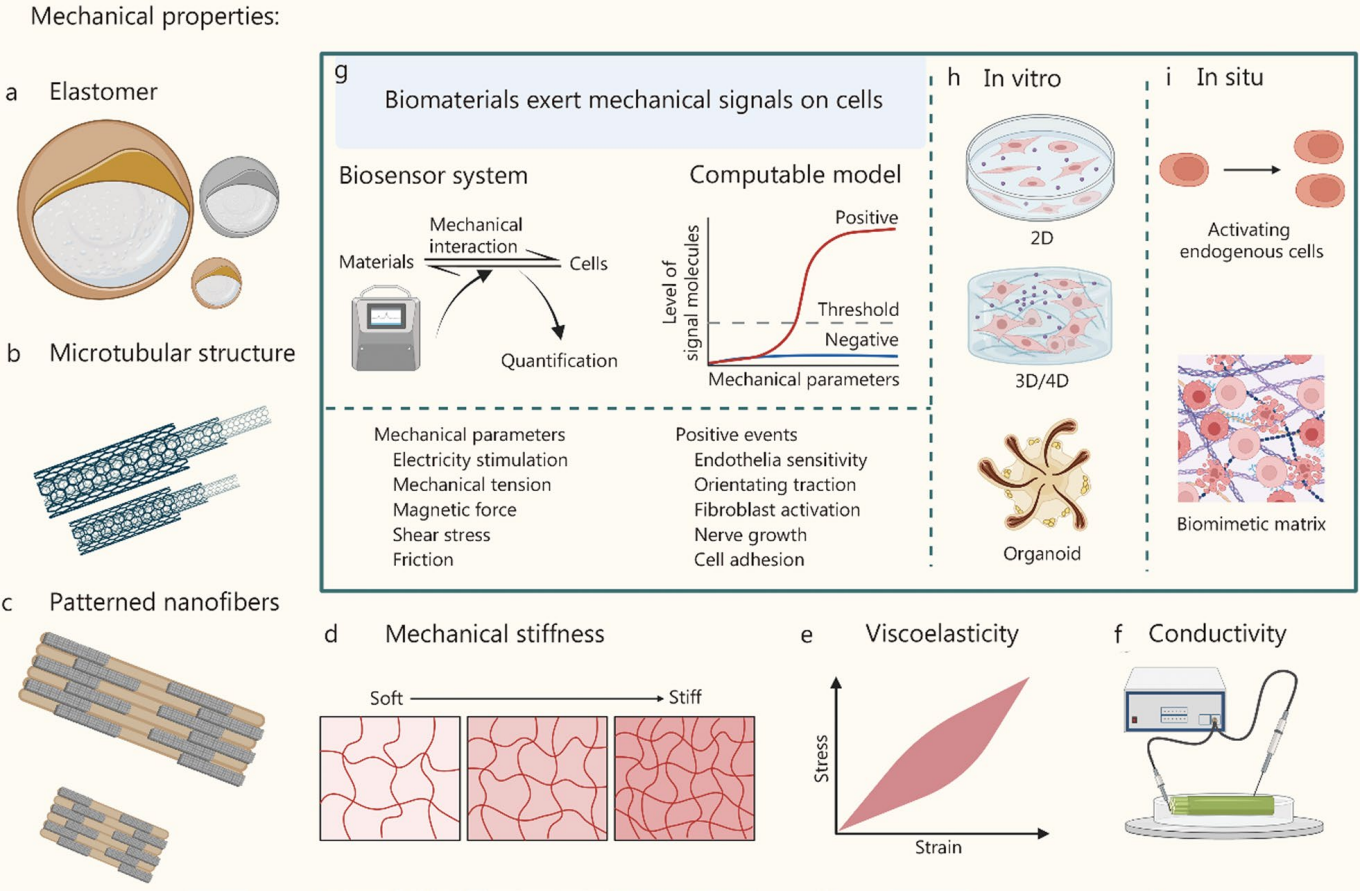
Biomaterial-based mechanical facilitation for scarless wound healing. Mechanical signals from biomaterials with specific physical properties are transformed to computable parameters by biosensor system. **a** Elastomer generates mechanical forces of different magnitudes and directions by stretching and contracting. **b** The hollow design of the microtubule structure allows for the mimicking of blood vessels and the inner wall simultaneously detects the velocity of the liquid as it flows through and the magnitude of the shear stress. **c** Patterned surface directs the growth of elongated tissue and it can mimic various two-dimensional (2D) structure of the matrix in vivo. **d** Surface with different stiffness renders cells different abilities to move. This is related to the stromal environment in which cells are physiologically located. **e** Viscoelasticity gives biomaterials solid-like properties such as elasticity, strength, and consequential stability as well as liquid-like properties such as flow properties that vary with time, temperature, load magnitude, and rate. This trait is more similar to the physiological situation. **f** Electrical conductivity is prevalent in living organisms, such as the resting and action potential of membrane potential. Biomaterials with electrical conductivity can be used to detect and simulate the required electrical signals within body. **g** Biomaterials and mechanical signals for cells: biosensor systems translate biomaterial-based mechanical signals into computable parameters like electricity stimulation, mechanical tension, magnetic force and friction. These quantitative parameters are then correlated with cell behaviors of interest (e.g., orienting traction, fibroblast activation, nerve growth, cell adhesion) through mathematical models. **h**–**i** Finally, precise regulation of cellular behavior can be achieved through biomaterial-based mechanical regulation whether in vitro (e.g., multidimensional culture of cells) or in situ (e.g., activating endogenous cells). Created with BioRender.com

Scars are characterized by excessive collagen deposition, which is a mechanical cue-dependent process. Elastomeric materials are reported to alter collagen deposition and arrangement mechanically, and thus, they have emerged as promising candidates to prevent scar formation [146] (Fig. 4a). Previous studies have revealed that collagen deposition and arrangement are influenced by the magnitude and direction of skin stretching tension-caused mechanical forces [147]. Collagen fibers prefer to align in the direction of mechanical forces, inspiring the development of elastomeric materials to regulate tension mechanically for scar prevention [147, 148]. Elastomeric materials are mainly fabricated based on various natural and synthetic polymers, and their deformability enable them to offload tension within the wound, or change tension direction [149], which provides the mechanism basis for applying elastomeric materials to regulate collagen. Currently, it has been reported that elastomeric materials (e.g., silicone gel, poly fibers and electro-spun micro-fibrous scaffolds) can regulate collagen deposition and arrangement through a diversity of molecular biology events. For instance, traditional silicone gel sheeting has been used for an extended period to prevent and treat hypertrophic and keloid scars [150, 151]. Silicone surfaces can interact with collagen and further lower the effective concentration of collagen monomers available to form fibrils. Besides, because the silicones can locally act as another “binding partner” in the fibrosis process in vivo, that interaction also leads to conformational changes in the collagen molecules [152]. Poly(3-hydroxybutyrate-co-3-hydroxyvalerate) (PHBV) fibers, as a widely used viscoelastic material, have the ability to regulate collagen expression level and increase the ratio of collagen I/collagen III, resulting in the inhibition of skin scar [153]. Electro-spun nanofibrous PHBV meshes, characterized with elastic fibrous networks and high porosity, reduce collagen deposition through blocking fibroblast-myofibroblast transition, lowering pro-fibrosis factor TGF-β1, and increasing anti-fibrosis factor TGF-β3. In addition, healed skin treated with electro-spun nanofibrous PHBV meshes is softer and more elastic. This highlights the versatility and effectiveness of elastomeric biomaterials in promoting scarless wound healing and skin regeneration [7]. Similar results are also observed in electro-spun micro-fibrous scaffolds crafted from the copolymer poly(L-lactide-co-ε-caprolactone), and that decreases scar formation [154]. The upgrade in fabrication methods of elastomeric material has expanded the possibilities for using elastic materials in scar treatment and prevention. One notable example is that photo-crosslinking technique endows elastomeric materials ultraviolet light-responsiveness. Utilizing riboflavin’s biophoton sensitivity, a photocurable silk fibroin hydrogel is fabricated. In this process, riboflavin is triggered by ultraviolet light to generate active oxygen radicals, which induces chemical crosslinking of amino, phenol, and other groups within the silk fibroin macromolecules, resulting in the formation of a photocurable hydrogel. These hydrogels exhibit remarkable resilience after compression and show satisfactory cytocompatibility, making them promising candidates to optimize collagen deposition [155]. Drawing inspiration from the nucleophilic Michael addition reaction, which involves the reaction between nucleophiles and activated alkynes, researchers have successfully created an easily processable elastomeric polyamide material with shape memory properties. This innovative material can mimic skin deformation well, so it has potential to regulate collagen deposition and arrangement [156]. By integrating 3D printing technology, researchers have developed a faster and more energy-efficient approach to producing a robust ordered mesoporous elastomer. This elastomer exhibits an ordered nano-microstructure and a molecular multinetwork, which together confers hierarchical toughening properties. Importantly, it maintains excellent stiffness and elongation, comparable to commonly used engineering elastomers like silicone and vulcanized rubber [157]. Furthermore, natural materials such as elastin and resilin, which are highly elastomeric, open up additional possibilities for developing elastomeric materials with unique properties and applications in various fields [158]. In summary, advances in elastic materials will facilitate better application in the regulation of collagen deposition and arrangement to prevent scar.

#### Tubular biomaterials

Considering that aberrant neovascularization can hinder scarless healing, there is a growing interest in achieving a well-organized vascular network rather than a disorganized vascular cluster through tubular biomaterials [159]. The organization and alignment of endothelial cells are pivotal for the formation of lumen-like structures and the development of well-structured blood vessels. A previous study has devised biodegradable conduits for the reconstruction of lymphatic vessels by facilitating the alignment and migration of lymphatic endothelial cells [94]. The formation of vessels is influenced by the balance between the magnitude of cell traction and the apparent stiffness of surrounding gel. In addition, vessel formation is less likely to occur in a very soft environment, highlighting the significance of the mechanical properties of surrounding tissue in regulating vascular network development [160]. Tubular biomaterials with suitable stiffness play a role in regulating the delicate balance between the growth and regression of blood vessels, and therefore, indicate a viable strategy to direct the well-organized neovascularization (Fig. 4b, d, e). In this strategy, shear stress is a crucial mechanical parameter which refers to the frictional force exerted by the flowing blood on the vascular endothelium, occurring parallel to the inner wall of blood vessels. The mechanical stimulus by shear stress can promote the development and remodeling of blood vessels, enhancing the process of neovascularization in tissues and contributing to their overall health and functionality [161–163]. An example is the use of a tubular collagen scaffold with shape memory for repairing tubular organs. This scaffold exhibits reversible expansion in response to increased luminal pressure and shear stress which further provides suitable attaching surface for endothelial cells [159]. This shear stress-responsive tubular collagen scaffold holds promise for well-organized neovascularization within wound. In addition, pre-implantation of cells into a specific tubular microfluidic system can promote the formation of blood vessels in vitro and normal vascular function [164]. In this setup, blood circulation flows through the lumen of these microtubes and shear stress occurs. Simultaneously, endothelial cells are carried by the blood flow and settle randomly along the inner surface of the microtube. These endothelial cells sense the shear stress generated by the flowing blood, leading to enhanced neovascularization. Tubular structures can be produced through various fabrication methods. Polymers such as polylactide-polycaprolactone, polylactide-co-glycolide, and polyhydroxyethyl methacrylate can be electro-spun to create tubular vascular grafts to promote angiogenesis and tissue regeneration [165]. Compared with synthetic materials, natural biopolymers are more conducive to cell attachment, migration, and proliferation when used as scaffolds. A novel biomimetic silk fibroin tubular scaffold crosslinked by poly(ethylene glycol) diglycidyl ether (PEG-DE) consists of three-layer structure: a regenerated SF intima, a silk braided media, and a regenerated SF adventitia. This silk fibroin tubular scaffold forms a porous layered tube with superior mechanical, permeable and cell adhesion properties that are beneficial to vascular regeneration [166]. Other forms of tubular materials include chitosan-carbon nanotube in the form of tubular hydrogel based on electrodeposition, tubular scaffold with circumferentially aligned nanofibers and so on [167, 168]. Collectively, various novel tubular biomaterials can be utilized to direct neovascularization through well-organized endothelial cells.

#### Specific-patterned biomaterials

In order to improve nerve restoration for better skin functional regeneration, specific-patterned nerve guidance conduits (NGCs) are employed to bridge the gap between the proximal and distal nerve stumps within lesions [169, 170]. These NGCs can be designed with diverse topographic features and provide appropriate mechanical environment for the growth of nerve tissue [171] (Fig. 4c–f). Plenty of evidences show that nerve outgrowth is mechanosensitive. Axon elongation can be induced by stretch forces [172]. Schwann cells sensing magnetic force-based mechanical stimulation accelerates peripheral nerve regeneration [8]. Electrospinning and micropatterning are commonly used technologies to create specific topographical features in NGCs. Electro-spun fibrous scaffolds are particularly appealing due to their high surface area-to-volume ratios, which promote cell attachment and growth. Micropatterning NGCs are characterized by smooth inner surfaces and longitudinal nanogrooves on the fiber surface. Schwann cells on anisotropic PA hydrogel micropatterned with aligned ridge/groove structures show obvious alignment growth and elongated neurites than on flat hydrogel [95, 173]. In addition, silk protein and polycaprolactone have also been used to construct composite anisotropic topography in the repair of peripheral nerve injury in adult rats [101, 169]. The height and angle of the groove could also influence neurites elongation. When culturing murine embryonic cortical neurons on micro-grooved platforms with a depth of 2.5–69.0 μm, axons are found to cross over the shallow grooves of 2.5 or 4.6 μm, whereas grooves of 22.0 or 69.0 μm cause the axons to turn after contact [174]. Except for aligned nanofibers and ridge/groove structures, pillars/posts, nanorough surfaces and surface stiffness are all common strategies used to modify the surface of NGCs [175]. For example, surface stiffness affects dorsal root ganglion (DRG) proliferation and differentiation in that soft hydrogel promotes the elongation of DRG’s axon by upregulating gene expression of *Ntn4* and *Unc5B* [176]. Despite the positive effect of topography on nerve tissue, it is difficult to achieve the desired results by solely relying on the topographic features of NGCs. Therefore, numerous biomolecules such as fibronectin, laminin, peptide, and growth factor have been immobilized in NGCs via physical adsorption, covalent grafting, or chemical crosslinking for accelerating nerve regeneration [101]. To sum up, specific patterned biomaterials act in concert with biochemical cues to enhance or improve the efficiency of nerve restoration.

#### Immunomodulatory biomaterials

Macrophages and neutrophils play a prominent role in responding to biomaterials and this inspire the development of immunomodulatory biomaterials for scarless wound healing [177]. Biomaterial surface, topography, wettability, and stiffness have been proved to regulate phenotype of macrophages and neutrophils. Rough surface produces more friction which promotes monocytes migration from the bloodstream into the wound area [178, 179] (Fig. 3e). Substrates with varying stiffness can exert mechanical forces on cells. For instance, when exposed to PA gels coated with fibronectin, macrophages on stiffer substrates exhibit an eightfold larger spreading area and a faster proliferation rate compared to those on softer substrates [96]. Similarly, using engineered 3D fibrillar matrices with natural biopolymers collagen I and glycosaminoglycans to mimic matrix with changing stiffness, macrophages preferentially turn to pro-inflammatory phenotype on stiffer substrate [97]. Ion channel Piezo1 can also be activated by cyclic stress to regulate macrophage polarization [180, 181]. As for the surface roughness and wettability, increased surface wettability had a stronger macrophage activation effect than increases in roughness [182]. Desirable cell and tissue response to an implanted biomaterial with specific topography can be achieved. For example, IL-1β produced by macrophages on expanded polytetrafluoroethylene with 4.4 μm pore (4.4-ePTFE) is 15 times higher than that from macrophages on non-porous PTFE [183]. Nanoscale surface topography of titanium (Ti) enhances the inflammation-related M1 phenotype in J774.A1 macrophages [184]. Fiber diameter of electro-spun poly(L-lactic) (PLLA) scaffolds influences the activation and protein secretion of RAW 264.7 macrophages in that nanofibrous PLLA scaffolds impair the inflammatory response mediated by macrophages when compared with films and micro-fibrous scaffolds [185]. Random fiber orientation topography promotes a pro-inflammatory signature in macrophages where higher percentage of CCR7^+^ cells appear on disorganized polycaprolactone nanofiber substrates, indicating a polarization shift towards a pro-inflammatory (M1-like) phenotype [186]. In a septic lung injury study, inhibiting Rho-kinase signaling with Y-27632 decrease the accumulation of neutrophils [187]. When exposed on smooth, rough, and rough-hydrophilic modified Ti surfaces, neutrophils show differences in protein secretion and NET formation. Specifically, anti-inflammatory cytokine (IL-4, IL-10) secretion is enhanced by rough hydrophilic Ti surfaces, and larger NETs are formed on rough surface [188]. Except for innate immunity, Griffin et al. [189] interestingly found that microporous annealed particle scaffolds could hasten the regenerative healing process by activating adaptive immune response. Therefore, immunomodulatory biomaterials show great potential in promoting scarless wound healing.

### Expanding stem or progenitor cell bank

Biomaterials regulate cellular plasticity through mechanical means, which will be an effective way to expand the stem and precursor cell bank [112, 190]. Various stem or progenitor cells are involved in the development of HF and SwG and they are influenced by mechanical forces from biomaterials [23, 191, 192], which is summarized in Table 2 [98, 193–202].

**Table 2:** Biomaterials for skin appendage regeneration

Cell	Material	Characteristic	Results	References
MSCs	Alginate-gelatin	Stiffness-dependent differentiation	Increasing differentiation of MSCs to SGCs	[98]
Akp2^+^Bmp6^+^ SKPs	Peptide hydrogel	Peptide nanofibers	Hair growth	[193]
CD200^+^, α6^+^ HFSC	Gelatin/alginate	Nanoscale biomimetic ECM	Maintaining the CD200^+^, α6^+^ HFSC population	[194]
In vivo	Bio-ceramic	Biofluid-absorbing bioactive sandwich structure	Enhanced hair growth	[195]
HFSCs	Silicone	Mesoporous nanoparticles loaded with Qu, Cu^2+^, and Zn^2+^	Regulating hair cycle	[196]
DPCs	EVAL membranes	Adhesive surface	Large-scale production of dermal papilla microtissues for HF regeneration	[197]
SGCs	Matrigel	Loaded with SGCs	Eccrine SwG	[198]
BM-MSCs	Matrigel	Three-dimensional structure	BM-MSCs differentiate into functional eccrine SGCs	[199]
SGCs	Gelatin	Microsphere loaded with EGF	SwG-like structure	[200]
In vivo	Calcium-alginate	Electrical signals triggered controllable formation	HF, sebaceous glands formation	[201]
In vivo	Wearable electronic device	Electricity stimulation	Higher HF density; longer hair shaft length	[202]

*MSCs* mesenchymal stem cells, *SKPs* skin derived precursors, *HFSCs* hair follicle stem cells, *DPCs* dermal papilla cells, *SGCs* sweat gland cells, *BM-MSCs* bone marrow-derived mesenchymal stem cells, *EVAL* poly(ethylene-co-vinyl alcohol), *ECM* extracellular matrix, *EGF* epidermal growth factor, *HF* hair follicle

The activation of hair follicle stem cells (HFSCs) is enhanced by decreasing mechanical forces from stiff niche in the context of both wound-induced HF neogenesis and aging [203, 204]. Due to the mechanosensitive characterization of HFSCs, an HFSC organoid culture system based on hydrogel has been modified to replicate the properties of young and aged basal membranes, with stiffness levels of 1–3 kPa and 5–6 kPa, respectively. This modification resulted in a noticeable increase in the activation of bivalent promoters, which controls crucial genes responsible for HFSC activation and self-renewal. It’s evident that mechanical forces originating from the niche can effectively suppress the activation of aged HFSCs [204]. Self-assembly peptides hydrogel enhances the proliferation of Akp2^+^Bmp6^+^ skin-derived precursors, and more importantly de novo hair genesis in mice [193]. Layer-by-layer self-assembly with gelatin/alginate is used to fabricate nanoscale biomimetic ECM for CD200^+^α6^+^ HFSCs population [194]. Natural minerals like silica, ceramic, are also used to fabricate 2D/3D support for HFSCs [195, 196]. Polymer biomaterials used to support hair follicular cells are poly(ethylene-co-vinyl alcohol) (EVAL) and PHBV. Compared with common PHBV medium, hair follicular epithelial cells (outer root sheath) and dermal cells (dermal sheath) cocultured with patterned PHBV nanofiber matrices exhibit greater HF regenerative and repair ability due to increased expression of collagen I, elastin, and α-SMA as well as more dermal papilla produced from dermal sheath [205]. EVAL membranes facilitate dermal papilla self-assembly into many compact spheroidal microtissues that are able to induce new HFs because adhesive surface is needed for quick cell expansion and a biomaterial with a lower adhesivity is required for self-aggregation [197].

Due to the presence of hair cycles, the above potential cells have the innate abundance within skin because they are reservoir for restoring the hair cycle. However, loss of SGCs, including luminal cells and myoepithelial cells, is hard to restore as they are terminally differentiated cells [206]. Autologous SwG-derived progenitors, induced pluripotent stem cells derived from somatic cells as well as fate conversed cells from different lineages are potential bank for forming SwGs [207]. The differentiation of mesenchymal stem cells to SGCs can be enhanced on 3D-bioprinted constructs made from alginate-gelatin blends with high stiffness [98]. Similarly, biomaterials with tunable mechanical properties are used to construct 3D even 4D (time) bionic ECM to induce differentiation of multiple stem cells (e.g., bone marrow-derived mesenchymal stem cells) toward SGCs thus increasing the abundance and quality of SwG progenitor cells [198, 199]. This illuminates a new direction for SwG regeneration based on the mechanical regulation of biomaterials. In addition, according to lineage-tracing, multipotent progenitors may include cells with high expression of keratin 15, CD29, or CD49f [207]. Although the expression of these markers has not yet been confirmed to be influenced by mechanical signals, it is a promising research direction. Biomaterial-based mechanical reprogramming of cell fate provides broader thinking. Self-adaptive biomaterials loaded with SwG regeneration-related signaling molecules (such as ectodysplasin-A, BMP-related molecules) can be used to achieve continuously release to activate endogenous cells like myoepithelial cells and coiled ductal cells thus promoting SwG regeneration in vivo [207]*.* To enhance the safety and efficiency of cell delivery, SGCs are cultivated on gelatin microspheres, and then this complex is implanted into an engineering skin construct to form SwGs [200]. What’s more, better biological reactor for SwG organoids formation in vitro is of great interest where mechanical signals from biomaterials are exerted to change cell fates. In the meantime, the stereoscopic structure provides cells with better living space and structural guidance. Collectively, biomaterials play an interesting role in SwG regeneration which deserve more explorations from micro mechanism to macro pro-regeneration efficiency.

### Supporting multiple appendages

**Fig. 5:**
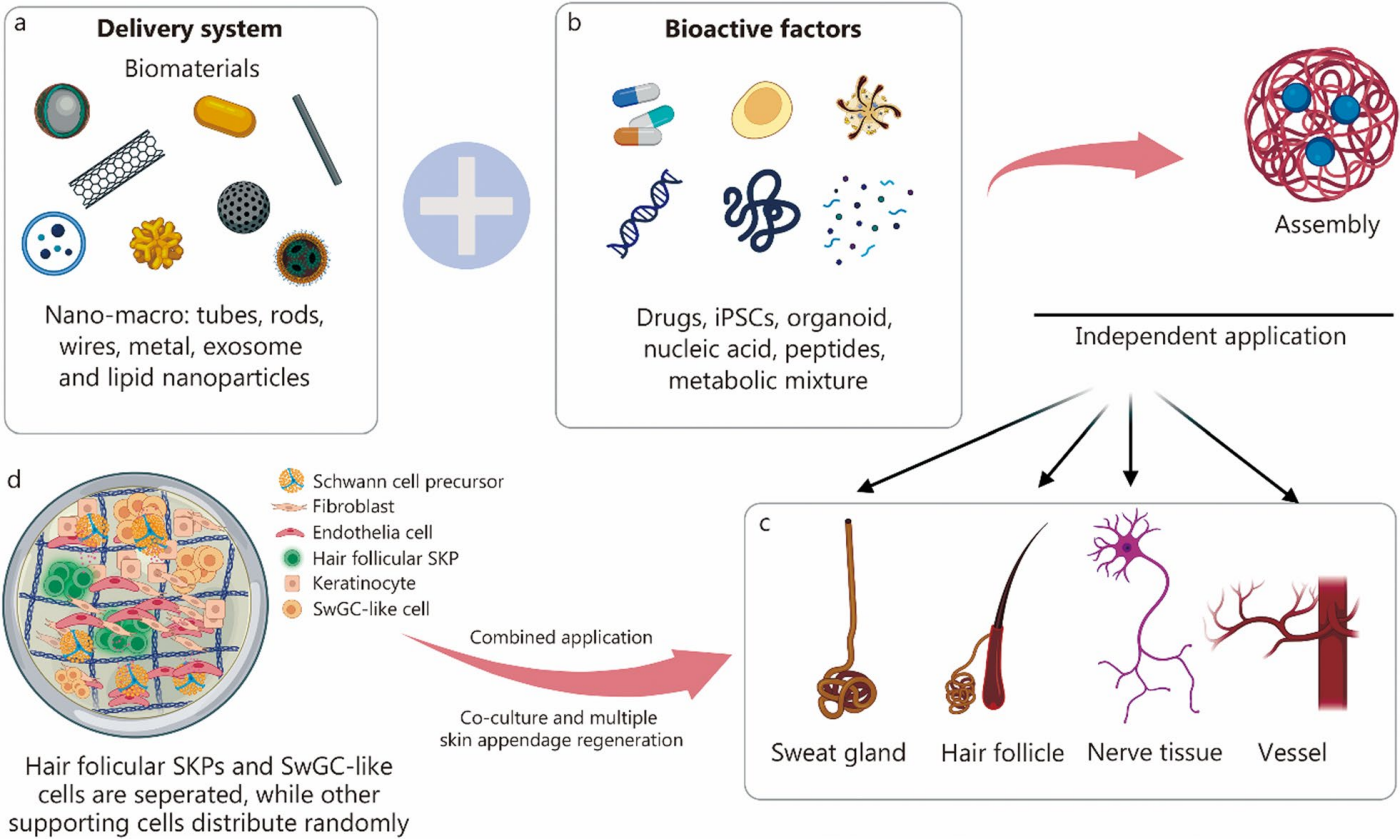
Biomaterials and regeneration of multiple appendages-an ideal skin unit. **a**, **b** Traditionally, biomaterials are used as carriers (diverse scales from nano- to macro-, e.g., tubes, rods, wires) to deliver bioactive factors (e.g., drugs, nucleic acid, peptides) or engineered cells (iPSCs) and organoids for tissue repair and regeneration. **c** In the field of skin wound repair, different combinations of biomaterials and bioactive ingredients have been used to regenerate sweat glands, hair follicles, nerve tissue, and vessel. However, a truly functional artificial skin requires a combination of in vitro and in vivo culture techniques for different cells and tissues. **d** It is promising to combine these assemblies into an integral construct to form a highly bionic skin unit. Biomaterials due to their variable physical properties can be used to create co-culture scaffolds for skin components, including vessel, nerve, hair follicles, sebaceous glands and sweat glands. In such a bionic skin, suitable conditions are separately established for the survival of various precursor cells (e.g., hair follicular SKPs, Schwann cell precursor, SwGC-like cells) with different regenerative signals to achieve co-culture of multiple skin appendages. Created with BioRender.com. iPSCs induced pluripotent stem cells, SKP skin derived precursor, SwGC sweat gland cell

Current in vivo or in vitro strategies for skin appendage regeneration can only restore single type of appendages (Fig. 5a–c). However, considering the native structure of skin where multiple appendages coexist, simultaneous regeneration of various skin appendages is attractive. The perfect repair of the skin must be accompanied by the regeneration of multiple appendages including HFs, SwGs, etc. Various plights hamper this ideal conceive. Biological problems trace back to the development of appendages. There are spatiotemporal antagonism signaling between SwGs and hair fate decision. Specifically, mesenchymal-derived BMP signal suppresses epithelial-derived sonic hedgehog (SHH) signal. BMP signal is for SwGs genesis while SHH signal is for hair. Therefore, hair genesis happens only when BMP is blocked, but it may be detrimental to SwG genesis. Naturally, starting signals for SwG genesis precede that for hair [208]. This chronological difference in timing limits artificial multiple-appendage regeneration. Booming biomaterials science may imply novel strategies. For example, biomaterials with separated chambers are expected to coculture SwG-like cells and hair follicular skin derived precursors in vitro through providing spatial freedom. Strategy that BMP signal is released first, followed by SHH signal can be achieved by smart biomaterials with sequential responsiveness to internal and external stimuli thus solving temporal conflicts. We propose a concept based on biomaterials called “co-culture and multiple skin appendage regeneration” where various appendages coexist (Fig. 5d). In the meantime, constructing highly biomimetic tissue engineering skin needs targeting dermal microcirculation, vascularized organoids as well as 3D bioprinting.

## Conclusion and outlook

In previous studies, in order to regenerate skin appendages, chemical method is always preferred. But accumulating evidences underscores the potential of targeted modulation of mechanical cues to enhance skin regeneration, promoting scarless repair by influencing the extracellular microenvironment and driving phenotypic transitions. In the meanwhile, the field of skin repair and skin appendage regeneration has witnessed remarkable advancements in the utilization of biomaterials with distinct physical properties. Therefore, we propose a global concept for scarless wound healing and skin appendage regeneration based on mechanical regulation combing with biomaterials. Notably, many studies remain confined to in vitro experiments, and even when transitioning to in vivo experiments, the subjects are typically limited to animals, which limits the translational application of biomaterials, making it less efficient. Therefore, it is crucial to shift more attention towards understanding the underlying mechanisms and principles. In the meantime, a difficult point in mechanical regulation is how to quantify the mechanical forces in biological effects (Fig. 4g–i). With the rise of biosensor technology, this problem is becoming increasingly soluble. Förster resonance energy transfer-based tension sensors have been extensively employed to measure forces and assess ECM stiffness at a small scale. This paves the way for more effective and targeted interventions in tissue regeneration and wound healing [209–211]. A low frequency Love wave sensor is used to detect 0.89–3.3 cP viscous change [212]. Machine learning-based image restoration and traction force microscopy are combined to understand relationship between cytoskeletal organization and ECM [213]. Biosensors play a crucial role in translating mechanical signals into computational models, offering a broader understanding of parameter quantification. Moreover, as we delve into the mechano-transduction pathway from FA to the nuclear lamina, it becomes evident that key players include, but are not limited to, integrins, the cytoskeleton, the LINC complex, and various associated proteins. In this intricate ‘relay race’, these components form a complex regulatory network that deserves further investigations. Scarless wound healing necessitates several essential factors, including high-quality re-epithelialization, reduced fibrosis, neovascularization, the restoration of innervation, and the regeneration of skin appendages. By combining computable mechanical parameters and the modification of key players in mechano-transduction, precise application of biomaterials can be achieved in scarless wound healing and skin appendage regeneration. This integrated approach holds promise for more effective interventions in tissue repair and regeneration.

## Data Availability

Not applicable.

## Abbreviations

2D: Two-dimensional
3D: Three-dimensional
ABPs: Actin-binding proteins;
α-SMA: α-Smooth muscle actin
BMP: Bone morphogenetic protein
CTGF: Connective tissue growth factor
CCM3: Cerebral cavernous malformations 3
CMCS: Carborxymethy chitosan
DRG: Dorsal root ganglion
ECM: Extracellular matrix
ECs: Endothelial cells
ELPs: Elastin-like polypeptides
EMT: Epithelial-mesenchymal transition
FAs: Focal adhesions
FAK: Focal adhesion kinase
GelMA: Gelatin methacryloyl
HDs: Hemidesmosomes
hES-MP: Human embryonic stem cell-derived mesenchymal progenitor cells
HFSCs: Hair follicle stem cells
HFs: Hair follicles
IFs: Intermediate filaments
IL: Interleukin
LATS1/2: Large tumor suppressor 1/2
LOX: Lysyl oxidase
LINC: Linker between nuclear-skeleton and cytoskeleton
MAPK: Mitogen-activated protein kinase
NETs: Neutrophil extracellular traps
NGCs: Nerve guidance conduits
PA: Polyacrylamide
PAI-1: Plasminogen activator inhibitor 1
PBA-PAMAM: Phenylboronic acid-bearing poly(amidoamine) dendrimer
PDMS: Polydimethylsiloxane
PDGF-B: Platelet-derived growth factor B
PEG-DE: Poly(ethylene glycol) diglycidyl ether
PHBV: Poly(3-hydroxybutyrate-co-3-hydroxyvalerate)
PI3K: Phosphatidylinositol-3-kinase
PLLA: Poly(L-lactic)
ROCK/MLC: RhoA-Rho kinase/myosin light chain
RREB1: RAS-responsive element binding protein 1
SGCs: Sweat gland cells
SHH: Sonic hedgehog
SMP: Shape memory polymer
SRM: Silicone rubber membrane
SRM-B: Silicone rubber membrane bilayer
ST2: Suppression of tumorigenicity 2 receptor
SwGs: Sweat glands
TGF: Transforming growth factor
Ti: Titanium
VEGF: Vascular endothelial growth factor
WISP1: WNT-inducible signaling pathway protein 1
YAP/TAZ: Yes-associated protein and transcriptional co-activator with PDZ-binding motif
ZEB1: Zinc finger E-box binding homeobox 1
ZEB2: Zinc finger E-box binding homeobox 2
